# Diversifying selection and color-biased dispersal in the asp viper

**DOI:** 10.1186/s12862-015-0367-4

**Published:** 2015-05-31

**Authors:** Sylvain Dubey, Valérie Zwahlen, Konrad Mebert, Jean-Claude Monney, Philippe Golay, Thomas Ott, Thierry Durand, Gilles Thiery, Laura Kaiser, Sylvia N Geser, Sylvain Ursenbacher

**Affiliations:** Department of Ecology and Evolution, Biophore Building, University of Lausanne, CH-1015 Lausanne, Switzerland; Section of Conservation Biology, Department of Environmental Sciences, University of Basel, St. Johanns-Vorstadt 10, CH-4056 Basel, Switzerland; Karch (Centre de coordination pour la protection des amphibiens et des reptiles de Suisse), Passage Maximilien-de-Meuron 6, CH-2000 Neuchâtel, Switzerland; Elapsoïdea, 21 chemin du Moulin, Bernex-Geneva, Switzerland; Wildensteinerstrasse45, 4416 Bubendorf, Switzerland; RD 118, 73200 Lyon, Césarches France; Rue du Pré de L’Ane, 805, 73000 Chambery, France

**Keywords:** Diversifying selection, Dispersal, Coloration, Reptile, Asp viper

## Abstract

**Background:**

The presence of intraspecific color polymorphism can have multiple impacts on the ecology of a species; as a consequence, particular color morphs may be strongly selected for in a given habitat type. For example, the asp viper (*Vipera aspis*) shows a high level of color polymorphism. A blotched morph (cryptic) is common throughout its range (central and western Europe), while a melanistic morph is frequently found in montane populations, presumably for thermoregulatory reasons. Besides, rare atypical uniformly colored individuals are known here and there. Nevertheless, we found in a restricted treeless area of the French Alps, a population containing a high proportion (>50%) of such specimens.

The aim of the study is to bring insight into the presence and function of this color morph by (i) studying the genetic structure of these populations using nine microsatellite markers, and testing for (ii) a potential local diversifying selection and (iii) differences in dispersal capacity between blotched and non-blotched vipers.

**Results:**

Our genetic analyses support the occurrence of local diversifying selection for the non-blotched phenotype. In addition, we found significant color-biased dispersal, blotched individuals dispersing more than atypical individuals.

**Conclusion:**

We hypothesize that, in this population, the non-blotched phenotype possess an advantage over the typical one, a phenomenon possibly due to a better background matching ability in a more open habitat. In addition, color-biased dispersal might be partly associated with the observed local diversifying selection, as it can affect the genetic structure of populations, and hence the distribution of color morphs.

**Electronic supplementary material:**

The online version of this article (doi:10.1186/s12862-015-0367-4) contains supplementary material, which is available to authorized users.

## Background

Color polymorphism is strongly correlated with the distribution of a species, its ecological niche width, as well as its genetic diversity. Indeed, color polymorphic species exhibit larger distributions, can use wider niches, and are genetically more diverse than monomorphic species [1-5]. In addition, polymorphic species seems to be more resilient to environmental modifications, an advantage which could have a non-negligible effect on their long-term survival (*e.g.* [5]). Such correlations may explain the numerous implications of coloration in processes of prey-predator interactions (*e.g.* aposematism or camouflage), thermoregulation, and behavior (e.g. [6-10]).

Particular coloration can confer advantages in specific conditions. For example, the occurrence of melanistic morphs in ectothermic vertebrates such as reptiles, has been documented in a large number of species, obviously for thermoregulatory reasons (e.g. [11-14]). In cold conditions, melanistic reptiles are able to increase their temperature faster than non-melanistic individuals of the same species, thus providing multiple advantages in terms of reproductive output, growth rate, survival or length of the activity period [10,15-18]. However, such benefits could be counterbalanced by a reduced level of crypsis or a lack of aposematic signaling. Therefore, melanistic individuals might experience a higher predation rate (e.g. [10,11,19]), possibly leading to increased stress and decreased foraging efficiency, which in turn could negatively impact their body condition (e.g. [10,20,21]). In addition, it may also indirectly affect the dispersal capacity of this morph, as non-cryptic dispersing individuals are more likely to be predated and never reach new habitats, leading to a color-biased dispersal. Few cases are known in which dispersal behaviors are color-specific, independently of the survival rate of dispersing individuals. For example, recent field studies focusing on the barn owl (*Tyto alba*) have shown that darker individuals disperse farther than paler individuals [22,23]. Similarly, Saino *et al.,* [24] found that barn swallow (*Hirundo rustica*) darker males were more likely to disperse. Such color-biased dispersals might deeply affect the population genetic structure of a species and its capacity to colonize new habitats, and might be partly associated to local diversifying selection.

For these reasons, particular color morphs may be under strong selection in a given habitat type. In order to test such a selection, an effective method is to compare color variation with the genetic differentiation (estimated using neutral genetic markers) of different populations to contrast the degree of adaptive variation and the degree of differentiation due to potential genetic drift [25-27]. The genetic divergence of neutral loci can serve as null-hypothesis to test against the adaptive divergence as an alternative [28,29].

**Figure 1:**
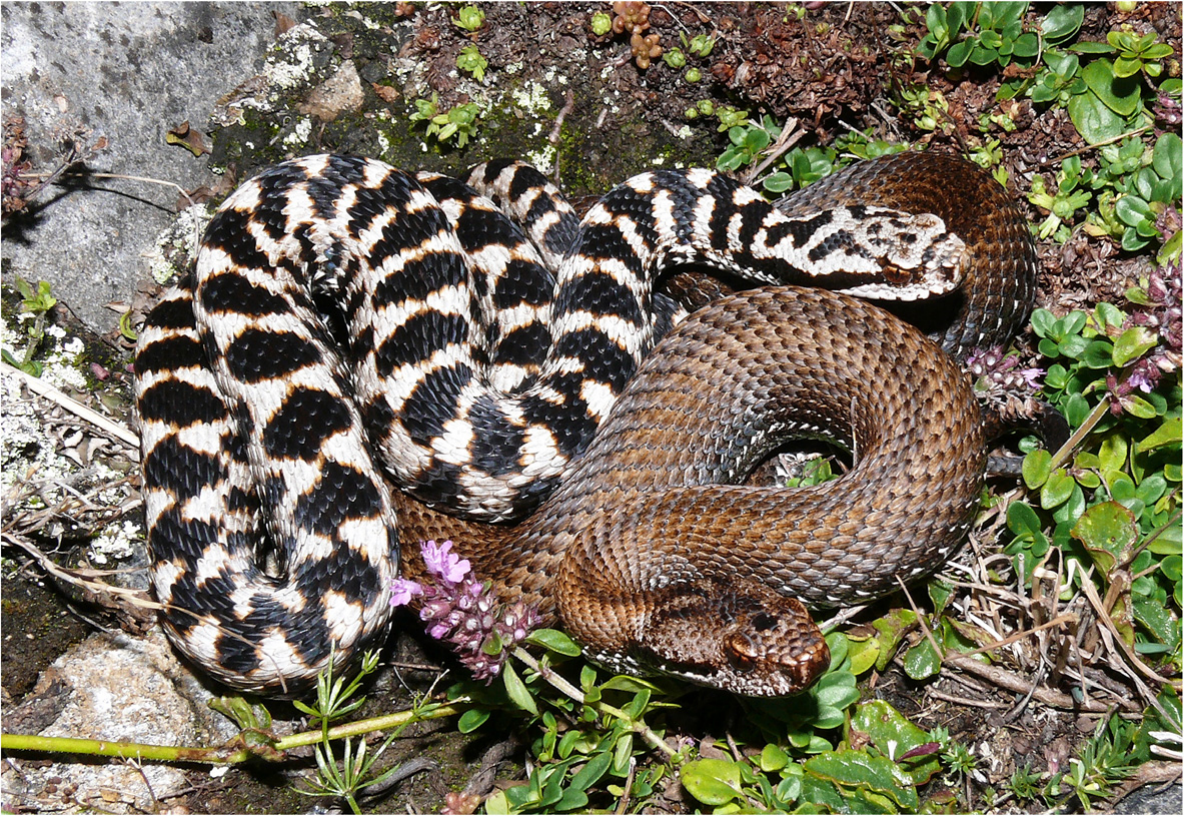
Examples of the color polymorphism present in two male asp vipers (*Vipera aspis*; blotched: left, non-blotched: right).

An appropriate model system to study this type of selection, and thus the evolution of color polymorphism, is the asp viper (*Vipera aspis*), which displays a high level of color variation in a large part of its distribution area (central and western Europe, from sea level to alpine areas). Blotched or zigzag morphs are common throughout *V. aspis* range; it is believed that these patterns have a cryptic function in European vipers, but also, once detected, reveal an aposematic signal to predators such as raptors [10,30,31]; Figure 1). In addition, a melanistic morph is frequently found in montane populations, particularly in the Swiss Alps, likely for thermoregulatory benefits [10,32]. Beside these two morphs, atypical non-blotched individuals (concolor with or without a middorsal line; Figure 1) are found in high proportions in the French Alps (; >50% in some Mont Blanc massif populations based on mark-recapture analyses; [33,34]), whereas this morph is rarely encountered in other regions and never in such proportions [33]). The adaptive function of this atypical coloration remains enigmatic, however the center of the area where non-blotched individuals are found in high frequencies is less wooded (i.e. characterized by large treeless areas) than its periphery, even at elevations as low as 1500 m above sea level (upper tree boundary is situated between 1800 - 2000 m). The principal aim of this study is to bring new insight into the presence of a large number of individuals with an atypical coloration in central Europe (Mont Blanc massif), by studying (i) the genetic structure of these populations using nine microsatellite markers, and testing for (ii) a potential local diversifying selection and (iii) differences in dispersal capacity between blotched and non-blotched individuals.

## Results

### F-statistics

We detected no evidence for null-alleles, scoring error due to stuttering or large allele dropout for the nine loci. In addition, we did not detect any significant linkage disequilibrium, or deviation from HWE within our 12 populations. For the nine microsatellite loci, the number of alleles per locus ranged from 3 to 32, with a total of 82 alleles across nine loci (Table 1). Expected heterozygosity within populations (H_E_) varied from 0.50 to 0.67 and observed heterozygosity (H_O_) varied from 0.52 to 0.65 (Table 2), whereas the allelic richness (AR) per population ranged from 3.0 to 4.33 (for six diploid individuals). We found a significant difference between populations with low and high proportions of non-blotched individuals (pop. 1-4 and 5-8, respectively) for H_E_ (Low = 0.62; High = 0.56; *P =* 0.048), but no significant differences for AR (Low = 3.81; High = 3.41; *P =* 0.099), H_O_ (Low = 0.60; High = 0.58; *P =* 0.27) and F_ST_ (Low = 0.069; High = 0.014; *P =* 0.15).

**Table 1:** **Mean F**_**is**_ **, expected and observed heterozygosities (H**_**E**_ **and H**_**0**_ **)** **, and number of alleles per locus**

	**F**_**is**_	**H**_**E**_	**H**_**0**_	**Alleles**
Vb-D17	0.05	0.91	0.87	32
Va-P70	−0.07	0.24	0.27	8
Va-P91	0.02	0.55	0.54	6
Va-P69	−0.18	0.73	0.86	8
Va-P35	0.06	0.70	0.66	7
Va-P81	0.13	0.70	0.61	4
Va-P25	0.04	0.58	0.56	7
Va-P20	−0.04	0.56	0.57	7
Vb-A8	0.00	0.37	0.37	3

**Table 2:** **Number of analysed individuals, proportion of non-blotched individuals (%), mean F**_**is**_ **, expected and observed heterozygosity (H**_**E**_ **and H**_**o**_ **)** **, mean F**_**st**_ **, and allele richness (AR) within populations and overall**

	**N**	**non-blotched (%)**	**F**_**is**_	**H**_**E**_	**H**_**o**_	**F**_**st**_	**AR**
Pop1	34	55.9	−0.02	0.58	0.59	0.05	3.60
Pop2	18	55.6	−0.068	0.57	0.61	0.05	3.44
Pop3	12	58.3	0.042	0.55	0.53	0.05	3.40
Pop4	14	64.3	−0.074	0.52	0.56	0.07	3.18
Pop5	15	26.7	−0.059	0.61	0.64	0.05	3.70
Pop6	6	0	−0.006	0.64	0.65	0.07	4.33
Pop7	8	0	0.012	0.61	0.61	0.07	4.12
Pop8	18	27.8	0.072	0.63	0.59	0.06	3.66
Pop9	18	5.6	0.038	0.67	0.64	0.06	4.00
Pop10	12	8.3	−0.009	0.60	0.61	0.07	3.86
Pop11	8	0	0.146	0.63	0.53	0.05	3.78
Pop12	7	14.3	−0.032	0.50	0.52	0.18	3.00
Overall	170	33.5	0.0035	0.59	0.59	0.07	4.04

### Gene flow & population structure

The genetic differentiations between populations (pairwise F_ST_) ranged from 0.00 to 0.24, with an overall F_ST_ value of 0.07 (*P* < 0.001; Table 3). In addition, the Mantel test indicated a genetic isolation by geographical distance (*r*^*2*^ = 0.325; *P =* 0.0001).

**Table 3:** **F**_**st**_ **(in bold: significant values; lower triangular matrix) and geographical distance (km) between pairs of populations (upper triangular matrix)**

	**Pop1**	**Pop2**	**Pop3**	**Pop4**	**Pop5**	**Pop6**	**Pop7**	**Pop8**	**Pop9**	**Pop10**	**Pop11**	**Pop12**
Pop1		2.46	3.59	6.50	5.36	7.47	10.08	5.02	6.40	6.51	5.95	9.92
Pop2	0.02		2.08	5.29	2.97	5.03	7.62	7.40	8.82	8.97	8.37	11.16
Pop3	0.00	0.02		3.22	3.77	5.31	7.38	8.52	9.81	9.67	8.83	9.95
Pop4	0.02	0.02	0.00		6.09	6.75	7.77	11.05	12.15	11.71	10.66	9.14
Pop5	**0.04**	**0.03**	0.03	**0.05**		2.15	4.90	10.07	11.55	11.83	11.31	13.70
Pop6	**0.07**	**0.06**	**0.08**	**0.13**	0.02		2.81	12.22	13.69	13.96	13.40	15.18
Pop7	**0.06**	**0.03**	**0.07**	**0.07**	0.01	0.02		14.94	16.40	16.59	15.95	16.79
Pop8	**0.05**	**0.06**	**0.06**	**0.06**	**0.06**	**0.09**	**0.08**		1.51	2.38	2.91	10.59
Pop9	**0.06**	**0.06**	**0.07**	**0.09**	**0.06**	**0.05**	**0.07**	**0.02**		1.40	2.50	10.54
Pop10	**0.06**	**0.07**	**0.07**	**0.10**	0.05	0.02	0.06	**0.06**	0.05		1.28	9.26
Pop11	0.04	0.04	0.02	0.06	0.04	0.06	0.07	0.00	0.01	0.04		8.04
Pop12	**0.18**	**0.19**	**0.18**	**0.20**	**0.19**	0.21	0.24	**0.14**	**0.14**	**0.24**	**0.13**	

**Figure 2:**
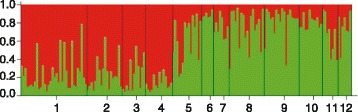
Summary plot of the individual assignment results of the Structure analyses (K = 2; Hubisz *et al.* [57]).

Following the method of Evanno *et al.* [35], the software Structure revealed two clusters within our dataset. Individuals of populations 1-4 mainly belonged to the first cluster, whereas individuals of populations 5-12 were more frequently assigned to the second cluster (Figure 2). Interestingly, populations of the first cluster are also those exhibiting the highest proportion of non-blotched individuals (56-64%) and have a central geographical distribution compared to the other sampled populations (5-12).

The BayesAss analysis suggested that recent exchange of migrants occurred between some populations, with a maximum value of m = 0.20 (std: 0.03) from population 1 into 4. In addition, the evaluation of gene flow revealed that it was asymmetrical between most populations (i.e. when standard deviations of gene flow between two populations did not overlap; see Additional file 1).

### Color and sex-biased dispersal analyses

**Figure 3:**
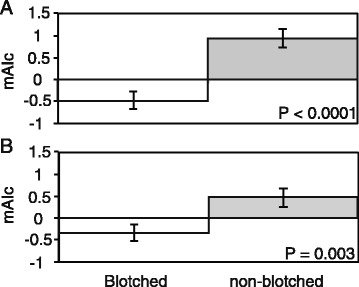
Mean Assignment Index correction (mAIc) values for blotched and non-blotched snakes considering all **(A)** or strictly polymorphic populations **(B)**.

Considering individuals from all or strictly color polymorphic populations (i.e. excluding pops 6,7, and 11), we found significant differences (Mann–Whitney U-test: Z = 4.27, *P* < 0.0001 and Z = 2.99, *P* = 0.003, respectively) between the mAIc of non-blotched (all populations: mean = 0.939; SE = 0.215; polymorphic populations: mean = 0.477; SE = 0.215; Figure 3) and blotched individuals (all populations: mean = -0.480; SE = 0.197; polymorphic populations: mean = -0.340; SE = 0.178), the latter having lower mAIc values, meaning that blotched individuals are dispersing the most. In addition, we found no significant differences between sexes overall (*P* = 0.12; males: mean = 0.250; SE = 0.199; females: mean = -0.159; SE = 0.179), as well as within non-blotched individuals (*P* = 0.45; males: mean = 0.104; SE = 0.473; females: mean = -0.055; SE = 0.281). However, sex biased dispersal was significant within blotched individuals, with females dispersing more than males (*P* = 0.05; males: mean = 0.346; SE = 0.195; females: mean = -0.243; SE = 0.188).

### P_st_ vs F_st_

The mean P_st_ values for the color trait were 0.17 when estimated with FSTAT, or 0.099, 0.17, and 0.28 when estimated with various level of potential heritability, i.e. 1, 0.5, and 0.1 respectively. All P_st_ values were higher than the empirical 95^th^ percentile of the pairwise F_ST_ distribution (this study: 0.086) and hence were considered as extreme, meaning that the coloration differentiations between populations might evolve by selection and not by neutral processes. The Wilcoxon signed rank tests revealed that there are significant difference between P_ST_ calculated with FSTAT (S = -437.5; *P* < 0.001), and with heritabilities of 0.5 (S = -438; *P* = 0.002) and 0.1 (S = -484; *P* < 0.001), but not when the heritability was set at 1 (S = -197; *P* = 0.18).

## Discussion

Our genetic analyses revealed a local diversifying selection and a color-biased dispersal (blotched individuals disperse more than non-blotched individuals). The combination of these results has several implications.

It is not known if non-blotched individuals are more frequently attacked by their predators (mainly raptors) than blotched individuals, in contrast to melanistic individuals [19,30], but the results of the present study attest the occurrence of selection for the uniformly colored phenotype, which is common only in the centre of the studied area (considering the other populations of the Mont Blanc massif). Thus, we hypothesize that this phenotype has a local cryptic function and is consequently better adapted to its habitat, which is less wooded, respectively more open than surrounding areas and characterized by light-colored stones. Indeed, several birds of prey occur in our study area, such as the short-toed snake eagle (*Circaetus gallicus*), which is a specific snake predator and can have a strong impact on viper populations [36].

An alternative scenario explaining the observed results would be selection against the non-blotched phenotype outside the core region and no selection within it. The results obtained in this study are also in agreement with this hypothesis. Indeed, the mAIcs of blotched individuals were lower than those of non-blotched individuals, meaning than these latter may be poor dispersers, or that non-blotched dispersers may be unable to survive out of their restricted geographical range. Based on our investigation, it is currently impossible to untangle these two hypotheses. However, mark-recapture and telemetry studies should provide helpful information concerning potential differences in the dispersal capacities of the different color morphs. Moreover, studying the predation rate of color polymorphic decoys could highlight differences in detectability between color morphs.

Furthermore, we cannot exclude that the observed pattern might be due to a recent range expansion (coupled with founder effects) of asp vipers in the study area. Such event could result in a low genetic structure and in a non-random distribution of the different color morphs, as observed in this study. Nevertheless, given (i) the lack of differences in the genetic diversity (both for AR and H_O_) between monomorphic and polymorphic populations, (ii) the particularity of the habitat where non-blotched snakes are found and (iii) the observed differences in dispersal capacity between blotched and non-blotched individuals (which might be linked to behavioral differences or to different survival rates of dispersers), a scenario involving a diversifying selection is more likely.

In a more general context, this atypical coloration, showing a lack of blotched or zigzag patterns on the dorsum of individuals, has been described in other viperids such as the Seoane’s viper (*Vipera seoanei*), meadow viper (*Vipera ursinii*, S. Ursenbacher & J.-P. Baron, pers. comm.) or Latifi’s viper (*Montivipera latifii*, [37]), but its impact on individuals is still unknown. However, several studies focusing on colubrid snakes and a species of salamander highlighted behavioral differences between color morphs in term of aggressiveness and predator avoidance strategies (*Thamnophis ordinoides*: [38,39]; *Coluber constrictor*: [40]; *Plethodon cinereus*: [41]).

The few studies focusing on color variation and the genetic structure of populations all showed that diversifying selection occurred. For example, Manier *et al.* [42] detected diversifying selection of different ecotypes of garter snake *Thamnophis elegans* (in terms of coloration and scalation). Cox & Rabosky [43] found that strong selection promotes color polymorphism across spatial and temporal scales in the highly polymorphic ground snake (*Sonora semiannulata*). In birds, Antoniazza *et al.* [44] found that local adaptation maintains clinal variation in melanin-based coloration of European barn owls (*Tyto alba*). In addition, the analyses of contact zones between closely related gull species (genus *Larus*) showed that interspecific divergence in plumage melanism and orbital ring color, clearly exceeded neutral genetic differentiation [45]. Interestingly, Abbott *et al.* [46] showed that diversifying selection occurred in color-polymorphic damselfly (*Ischnura elegans*) populations in a given year, while two generations later (two years) population differentiation in morph frequencies fell behind neutral genetic differentiation. Consequently, it is consistent with a temporal heterogeneity in selection in these populations, meaning that selection might vary over time, where both spatial and temporal heterogeneities likely play an important role in promoting and maintaining polymorphism. In addition, in the Californian spider, *Theridion californicum*, characterized by at least eleven color morphs, genetic analyses of several populations revealed that such polymorphism is maintained through balancing selection, i.e. acting to maintain polymorphism across populations [47].

Overall, these studies suggested that observed intraspecific color variations in both vertebrates and invertebrates are often the result of a local adaptation and are not due to a random genetic drift. Therefore, observed color polymorphism in these studies is not neutral from an evolutionary point of view. In this respect, recent studies highlighted that the presence of intraspecific color polymorphism might increase the adaptive potential of a species hence its long-term survival and capacity to deal with environmental variations (e.g. [1]). As a consequence, the important color polymorphism found in the asp viper might be accountable for its unique capacity among reptiles to deal with a large number of habitat types, ranging from Mediterranean coastal areas to alpine regions (up to 2500m above sea level; [48]). Indeed, color morphs and their intrapopulational frequencies are tightly linked to geographical regions and habitat types (e.g. [10,33]). A recent field study highlighted intrapopulational sex-specific differences in body condition between melanistic and blotched *V. aspis* [10], melanistic females exhibiting higher body condition than blotched ones. These results were attributed to the importance of an efficient thermoregulation for females during gestation, and to higher rate of predation in melanistic males compared to blotched ones. Since males are actively searching for females during the breeding season, and are forced to move away from their shelter, their chance of being predated is greater than for females [10]. These results illustrated the complex role that coloration plays in ectothermic vertebrates, and how it can be involved in the evolution of such organisms.

## Conclusions

The presence of important color polymorphism within a species may provide more opportunities to adapt and cope with different environmental pressures [42], leading in turn to a potentially larger distribution area and a higher resilience. Even though the studied area presents a unique case in the asp viper, investigating the different environmental characteristics (biotic and abiotic) leading to the local selection of this particular pattern can be of major interest to understand i) the selection pressure on the dorsal coloration in ectothermic vertebrates ii) the speed of the morphological adaptation and iii) the importance of such phenotypic diversity within species.

## Methods

### Study site and tissue sampling

**Figure 4:**
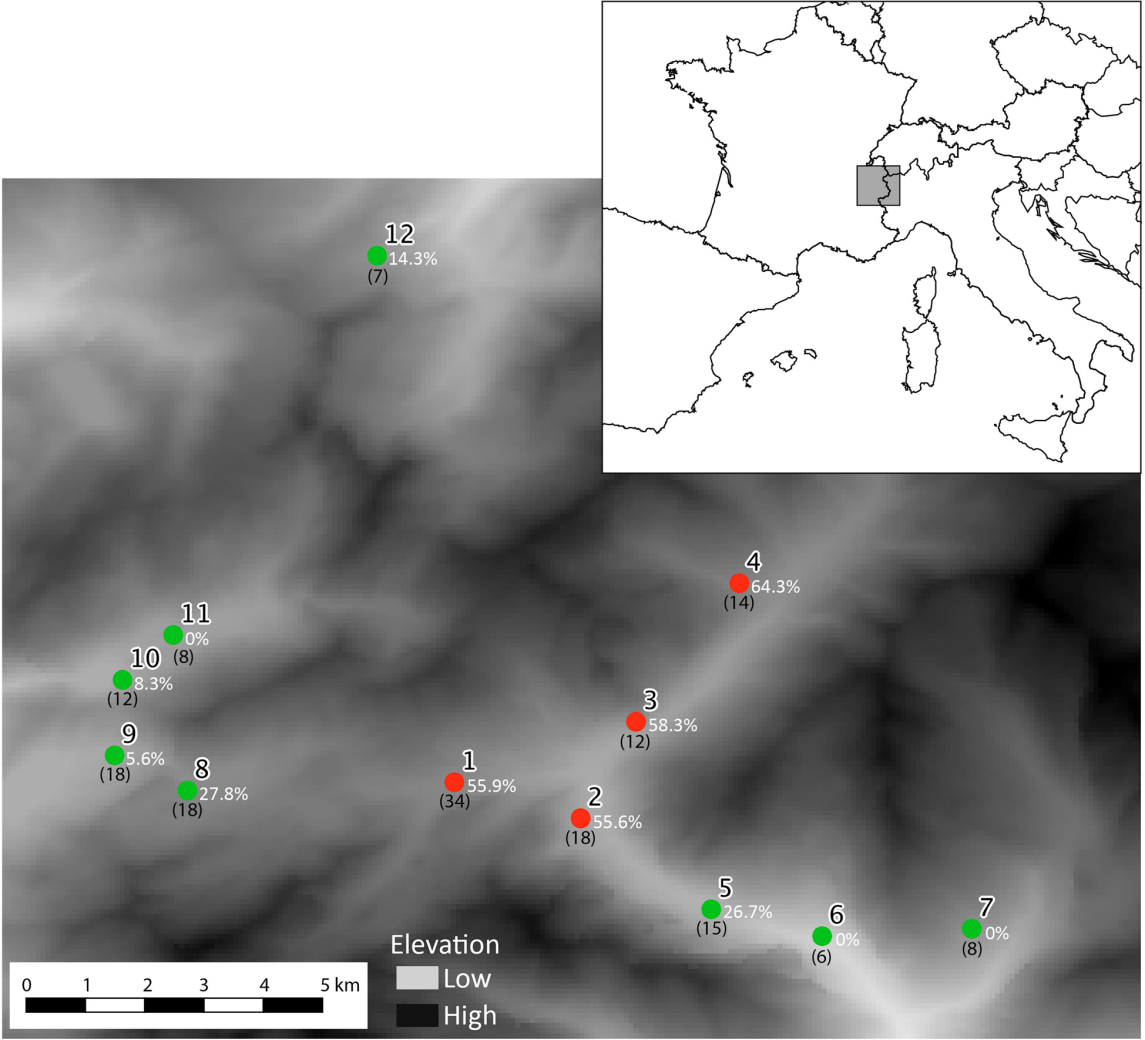
Distribution and proportion of non-blotched individuals of sampled populations. In red and green, populations mainly assigned to the first and second genetic cluster identified by the Structure analyses (Hubisz *et al.* [57]).

The study site is located in the French Alps (Mont Blanc massif), between 1’100 and 2’100 m above sea level. We collected a total of 170 samples from blotched (N = 113) and non-blotched (N = 57) snakes between 2006 and 2010. Based on their location, we grouped the samples into 12 populations (see Figure 4; in red, populations with a proportion of non-blotched individuals higher than 50%).

For each captured individual, we collected the coordinates either with a GPS or with the help of Google Earth v5.0 (Google, Mountain View, US), both methods allowing an accuracy of about 10 m. We collected DNA samples for the genetic analyses from blood, ventral scales, tip of the tail (stored in 90% ethanol prior to DNA extraction), and/or buccal swab. In order to avoid duplicate samplings, we took dorsal and head pattern pictures (dorsal and lateral view) for each specimen. Color morphs of individuals were determined in the field. Several persons scored the phenotype of snakes simultaneously and none of the 170 individuals used in the present study had an ambiguous phenotype. Indeed, blotched (characterized by a zigzag-like pattern) and atypical non-blotched individuals (light-colored individuals with an absence of zigzag-like pattern) were sufficiently different to avoid misclassifications (Figure 1).

### DNA extraction

We extracted DNA using the QIAGEN DNeasy® kit (QIAGEN, Hombrechtikon, Switzerland) according to the DNeasy® Blood & Tissue Handbook. In order to improve the quality of extractions, we modified a few steps: (i) overnight lysis was conducted for most samples, (ii) elution was performed twice using 100 μl of Buffer AE and finally (iii) incubation time was extended to 5 min so that a higher concentration of DNA was obtained. For buccal DNA sampling, the DNA extraction was also following the same protocol, except that swabs were placed into the DNeasy Mini spin column and centrifuged to remove the remaining liquid from them between steps 4 and 5 of the protocol provided by the manufacturer.

### Microsatellite analyses

Seven loci from *V. aspis* (Va-P70, 91, 69, 35, 81, 25, 20; [49]) and two loci from *V. berus* (Vb-A8 and Vb-D17; [50]) were amplified and scored. We performed PCR in an Eppendorf Mastercycler Gradient (Vaudaux-Eppendorf AG, Basel, Switzerland), with a final volume of 12 μl per reaction, using 2-4 μl of DNA extraction 1x PCR buffer, 2 mg/ml of Q solution, 0.2 mM dNTPs, 0.5 units of *Taq* Polymerase, and, depending on the amplified locus, 1.5 - 3.5 mM of MgCl_2_ (all reagents from QIAGEN) and 0.25 – 0.5 mM of each primer (see Additional file 1 for more details). Cycling conditions included 37 to 40 cycles of 95°C for 30 sec, 50 to 57**°**C (depending on the microsatellite locus) annealing temperature during 30 sec, 72**°**C for 45 sec, and a final extension of 72°C for 7 min (see [49] for more details). Then, we genotyped amplified products with an ABI3130xl genetic analyzer (Applied Biosystems) and visualized with PEAK SCANNER™ Software v1.0 (Applied Biosystems).

### F-statistics and genetic diversity parameters

First, we tested for the presence of null-alleles, scoring error due to stuttering and large allele dropout with MICRO-CHECKER v 2.2.3 [51]. We calculated the genotypic disequilibrium between loci in each sample based on 10,000 randomizations to check for linked loci. We tested deviations from Hardy–Weinberg equilibrium (HWE) within samples based on 10,000 randomizations. We estimated the Wright’s fixation indices for within-population deviation from random mating (F_IS_), as well as pairwise subpopulation differentiation (F_ST_), following Weir & Cockerham [52]. We computed deviations from random mating within populations (F_IS_) per locus and sample with a bootstrap procedure including 10,000 randomizations. We estimated expected (H_E_) and observed (H_O_) heterozygosities following the methods of Nei & Chesser [53] and allelic richness (AR) with FSTAT. In addition, we performed a Mantel test [54] with genetic distance (pairwise F_ST_) as the dependent variable and the distance between sites as explanatory variable. We carried out permutation tests in order to detect significant differences in allelic richness, expected (H_E_) and observed (H_O_) heterozygosities and F_ST_ indices among the populations with a high versus a low amount of non-blotched individuals (populations 1-4 and 5-12, respectively). We performed all summary statistics and tests using the software FSTAT Version 2.9.3.2 [55]. The critical *p*-value of 0.05 was adjusted using the Bonferroni correction [56] due to multiple comparisons.

### Structure of populations

We used the software Structure version 2.3.4 [57], a Bayesian model-based clustering method [58], to infer population structure and to assign individuals to populations. Based on allele frequencies, we used this MCMC simulation to assign a membership coefficient for each individual to each K populations. Ten runs of 600,000 iterations (the first 200,000 considered as burn-in) for K = 1–12 were performed including all individuals. Then, we defined the number of clusters that best fits our data set as described in Evanno *et al.* [35]. This approach compares the rate of change in the log probability of data between successive K and the corresponding variance of log probabilities.

### Unidirectional gene flow among populations

In order to quantify unidirectional migration rates (m) between populations, we used the software BayesAss 3.0.3 [59]. This Bayesian method relies on the tendency for immigrants to show temporary disequilibrium in their genotypes relative to the focal population, which allows their identification as immigrants or offspring of immigrants. After initial runs were conducted with variable values of deltaA, deltaM and deltaF in order to improve the acceptance levels, the best values were set to deltaA = 1, deltaM = 0.2 and deltaF = 1. For the final analysis, we used 3 x 10^6^ MCMC iterations, including a burn-in length of 3 x 10^5^ iterations.

### Sex and color-biased dispersal

We tested for sex- and color-biased dispersal in our populations, using GenAlEx 6.5 [60]. Then, we compared the mean of the corrected assignment index (mAIc; [61]) between sexes (males vs. females), as well as between colors (blotched vs. non-blotched individuals). With this statistical approach, residents tend to have higher mAIc values than immigrants.

### Comparison between genetic and morphological variation

A commonly used method to estimate population differentiation for a quantitative trait is a metric called Q_ST_, an analog of F_ST_, which calculates the genetic differentiation at neutral genetic markers. Because Q_ST_ calculation requires experimental estimates of additive genetic variances and since we did not estimate additive genetic variance for asp viper coloration, we will refer in this study to P_ST_ (phenotypic or pseudo-Q_ST_) rather than Q_ST_, as proposed by Saether *et al.* [62]. P_ST_ was estimated with two different methods. First, we coded the color as a locus having only two alleles, blotched individuals being homozygotes coded 11 at this locus and non-blotched individuals being homozygotes 22. Then, we used FSTAT to calculate the pairwise P_ST_ between populations. Second, we estimated pairwise P_ST_-values as a function of heritability (h^2^), within- ${\upsigma}_w^2$ and between-populations phenotypic variances (${\upsigma}_{\mathrm{b}}^2$; [29,63]):

$$ {\mathrm{P}}_{\mathrm{ST}} = \frac{\upsigma_{\mathrm{b}}^2}{\upsigma_{\mathrm{b}}^2+2{h}^2{\upsigma}_w^2} $$

We obtained the within- and between populations variances by extracting the mean squares (*MS*) with an ANOVA on color in JMP 10.0 (SAS Institute, Cary, NC, USA). Because the heritability of the color pattern is not known, we used three different values of heritability (0.1, 0.5 and 1). We estimated within-population variance $\left({\upsigma}_w^2\right)$ without bias by within-population MS, whereas between-population variance $\left({\upsigma}_{\mathrm{b}}^2\right)$ is calculated as follows:

$$ {\upsigma}_{\mathrm{b}}^2=\frac{M{S}_b-M{S}_w}{n_0} $$

where *MS*_*b*_ and *MS*_*w*_ are the within- and between-population MS and *n*_*0*_ is a weighted average of the sample size for each population comparison estimated following Sokal & Rohlf [64] as:

$$ {n}_0=\frac{1}{a-1}\left({\displaystyle \sum^n}{n}_i-\frac{{\displaystyle {\sum}_a}{n}_i^2}{{\displaystyle {\sum}_a}{n}_i}\right) $$

where n_i_ the number of individuals in the i^th^ population and *a* is the number of populations to be compared.

To test whether the coloration differentiations between populations evolved by neutral processes or selection, we compared P_ST_ values obtained for individual traits and with the two different methods with the distribution of pairwise F_ST_ values. As in Slavov *et al.* [65], we considered P_ST_ values as extreme when exceeding the empirical 95^th^ percentile of the F_ST_ distribution (in this study: 0.086). In addition, to check for significant difference between P_ST_ - and F_ST_-values, we performed a Wilcoxon signed rank test [66].

### Availability of supporting data

Microsatellite genotypes and phenotypes: Dryad doi: 0.5061/dryad.87478.

### Animal ethics

The samples have been taken with the authorizations of the local authorities (Autorisation préfectorale No. 2009-14; Direction de l’Administration Territoriale et de l’Environnement, Bureau de l’Environnement et du Développement Durable, Préfecture de la Savoie, 73018 Chambéry, France).

## Additional file

Additional file 1:
**Unidirectional migration-rate estimates within pairs of asp viper populations (in italic: standard deviation).**
