# Supplementary material for: Diversifying selection and color-biased dispersal in the asp viper
Source: BMC Evol Biol. 2015 May 31;15:99. doi: 10.1186/s12862-015-0367-4 (PMC4449969; doi:10.1186/s12862-015-0367-4)
Supplement: Additional file 1: — Unidirectional migration-rate estimates within pairs of asp viper populations (in italic: standard deviation). [file 12862_2015_367_MOESM1_ESM.docx]

**Additional file 1:** Unidirectional migration-rate estimates within pairs of asp viper populations (in italic: standard deviation). m[i][j] is the fraction of individuals in population i that are migrants derived from population j.

| m[1][1]: | 0.881 | *0.028* | m[1][2]: | 0.013 | *0.012* | m[1][3]: | 0.007 | *0.007* | m[1][4]: | 0.008 | *0.008* | m[1][5]: | 0.015 | *0.013* | m[1][6]: | 0.007 | *0.007* |
| --- | --- | --- | --- | --- | --- | --- | --- | --- | --- | --- | --- | --- | --- | --- | --- | --- | --- |
| m[2][1]: | 0.179 | *0.040* | m[2][2]: | 0.706 | *0.032* | m[2][3]: | 0.011 | *0.010* | m[2][4]: | 0.010 | *0.009* | m[2][5]: | 0.013 | *0.013* | m[2][6]: | 0.011 | *0.010* |
| m[3][1]: | 0.180 | *0.036* | m[3][2]: | 0.016 | *0.014* | m[3][3]: | 0.682 | *0.014* | m[3][4]: | 0.013 | *0.012* | m[3][5]: | 0.015 | *0.014* | m[3][6]: | 0.013 | *0.012* |
| m[4][1]: | 0.197 | *0.030* | m[4][2]: | 0.013 | *0.013* | m[4][3]: | 0.012 | *0.012* | m[4][4]: | 0.680 | *0.013* | m[4][5]: | 0.012 | *0.012* | m[4][6]: | 0.012 | *0.011* |
| m[5][1]: | 0.055 | *0.049* | m[5][2]: | 0.082 | *0.055* | m[5][3]: | 0.012 | *0.011* | m[5][4]: | 0.013 | *0.012* | m[5][5]: | 0.712 | *0.041* | m[5][6]: | 0.013 | *0.012* |
| m[6][1]: | 0.020 | *0.018* | m[6][2]: | 0.030 | *0.027* | m[6][3]: | 0.018 | *0.018* | m[6][4]: | 0.018 | *0.017* | m[6][5]: | 0.094 | *0.047* | m[6][6]: | 0.689 | *0.020* |
| m[7][1]: | 0.045 | *0.035* | m[7][2]: | 0.066 | *0.046* | m[7][3]: | 0.017 | *0.017* | m[7][4]: | 0.016 | *0.015* | m[7][5]: | 0.044 | *0.038* | m[7][6]: | 0.017 | *0.016* |
| m[8][1]: | 0.043 | *0.030* | m[8][2]: | 0.019 | *0.019* | m[8][3]: | 0.011 | *0.010* | m[8][4]: | 0.011 | *0.011* | m[8][5]: | 0.016 | *0.016* | m[8][6]: | 0.011 | *0.011* |
| m[9][1]: | 0.031 | *0.025* | m[9][2]: | 0.017 | *0.016* | m[9][3]: | 0.011 | *0.011* | m[9][4]: | 0.011 | *0.011* | m[9][5]: | 0.020 | *0.019* | m[9][6]: | 0.012 | *0.012* |
| m[10][1]: | 0.030 | *0.026* | m[10][2]: | 0.023 | *0.022* | m[10][3]: | 0.014 | *0.013* | m[10][4]: | 0.014 | *0.013* | m[10][5]: | 0.021 | *0.021* | m[10][6]: | 0.014 | *0.014* |
| m[11][1]: | 0.057 | *0.038* | m[11][2]: | 0.021 | *0.021* | m[11][3]: | 0.017 | *0.016* | m[11][4]: | 0.017 | *0.016* | m[11][5]: | 0.019 | *0.018* | m[11][6]: | 0.016 | *0.015* |
| m[12][1]: | 0.030 | *0.025* | m[12][2]: | 0.019 | *0.018* | m[12][3]: | 0.017 | *0.016* | m[12][4]: | 0.018 | *0.017* | m[12][5]: | 0.019 | *0.019* | m[12][6]: | 0.017 | *0.016* |

| m[1][7]: | 0.008 | *0.008* | m[1][8]: | 0.012 | *0.011* | m[1][9]: | 0.016 | *0.013* | m[1][10]: | 0.017 | *0.015* | m[1][11]: | 0.008 | *0.007* | m[1][12]: | 0.009 | *0.009* |
| --- | --- | --- | --- | --- | --- | --- | --- | --- | --- | --- | --- | --- | --- | --- | --- | --- | --- |
| m[2][7]: | 0.010 | *0.009* | m[2][8]: | 0.014 | *0.013* | m[2][9]: | 0.013 | *0.013* | m[2][10]: | 0.011 | *0.011* | m[2][11]: | 0.011 | *0.011* | m[2][12]: | 0.012 | *0.012* |
| m[3][7]: | 0.013 | *0.013* | m[3][8]: | 0.014 | *0.014* | m[3][9]: | 0.014 | *0.013* | m[3][10]: | 0.016 | *0.015* | m[3][11]: | 0.013 | *0.013* | m[3][12]: | 0.014 | *0.013* |
| m[4][7]: | 0.012 | *0.012* | m[4][8]: | 0.012 | *0.011* | m[4][9]: | 0.014 | *0.013* | m[4][10]: | 0.013 | *0.012* | m[4][11]: | 0.012 | *0.011* | m[4][12]: | 0.012 | *0.011* |
| m[5][7]: | 0.013 | *0.013* | m[5][8]: | 0.018 | *0.018* | m[5][9]: | 0.025 | *0.021* | m[5][10]: | 0.033 | *0.022* | m[5][11]: | 0.011 | *0.011* | m[5][12]: | 0.014 | *0.014* |
| m[6][7]: | 0.020 | *0.019* | m[6][8]: | 0.020 | *0.018* | m[6][9]: | 0.023 | *0.021* | m[6][10]: | 0.032 | *0.024* | m[6][11]: | 0.018 | *0.017* | m[6][12]: | 0.019 | *0.017* |
| m[7][7]: | 0.688 | *0.019* | m[7][8]: | 0.021 | *0.019* | m[7][9]: | 0.021 | *0.019* | m[7][10]: | 0.029 | *0.025* | m[7][11]: | 0.017 | *0.016* | m[7][12]: | 0.018 | *0.017* |
| m[8][7]: | 0.011 | *0.011* | m[8][8]: | 0.788 | *0.048* | m[8][9]: | 0.040 | *0.036* | m[8][10]: | 0.024 | *0.023* | m[8][11]: | 0.012 | *0.012* | m[8][12]: | 0.014 | *0.014* |
| m[9][7]: | 0.012 | *0.011* | m[9][8]: | 0.092 | *0.051* | m[9][9]: | 0.753 | *0.047* | m[9][10]: | 0.017 | *0.017* | m[9][11]: | 0.011 | *0.010* | m[9][12]: | 0.014 | *0.013* |
| m[10][7]: | 0.015 | *0.015* | m[10][8]: | 0.049 | *0.045* | m[10][9]: | 0.055 | *0.045* | m[10][10]: | 0.735 | *0.036* | m[10][11]: | 0.013 | *0.013* | m[10][12]: | 0.016 | *0.016* |
| m[11][7]: | 0.017 | *0.015* | m[11][8]: | 0.059 | *0.042* | m[11][9]: | 0.027 | *0.024* | m[11][10]: | 0.040 | *0.033* | m[11][11]: | 0.687 | *0.019* | m[11][12]: | 0.024 | *0.020* |
| m[12][7]: | 0.018 | *0.016* | m[12][8]: | 0.019 | *0.018* | m[12][9]: | 0.021 | *0.020* | m[12][10]: | 0.018 | *0.017* | m[12][11]: | 0.018 | *0.016* | m[12][12]: | 0.787 | *0.036* |
